# Supplementary material for: Giant thermal hysteresis in Verwey transition of single domain Fe3O4 nanoparticles
Source: Sci Rep. 2018 Mar 23;8:5092. doi: 10.1038/s41598-018-23456-6 (PMC5865112; doi:10.1038/s41598-018-23456-6)
Supplement: Supplementary file 1 — Supplementary Information [file 41598_2018_23456_MOESM1_ESM.pdf]

Supplementary Information

Giant thermal hysteresis in Verwey transition of single domain Fe_3O_4 nanoparticles

Taehun Kim[1,2*], Sumin Lim[3*], Jaeyoung Hong[4,5*], Soon Gu Kwon[4,5*], Jun Okamoto[6], Zhi Ying Chen[7], Jaehong Jeong[1,2], Soonmin Kang[1,2], Jonathan C. Leiner[1,2], Jung Tae Lim[8], Chul Sung Kim[8], Di Jing Huang[6,7], Taeghwan Hyeon[4,5], Soonchil Lee[3], and Je-Geun Park[1,2,#]

[1]Center for Correlated Electron Systems, Institute for Basic Science, Seoul 08826, Korea

[2]Department of Physics & Astronomy, Seoul National University, Seoul 08826, Korea

[3]Department of Physics, Korea Advanced Institute of Science and Technology, Daejeon 34141, Korea

[4]Center for Nanoparticle Research, Institute for Basic Science, Seoul 08826, Korea

[5]School of Chemical and Biological Engineering, Seoul National University, Seoul 08826, Korea

[6]National Synchrotron Radiation Research Center, Hsinchu 30076, Taiwan

[7]Department of Physics, National Tsing Hua University, Hsinchu 30013, Taiwan

[8]Department of Physics, Kookmin University, Seoul 02703, Korea

* Authors with equal contribution

# Corresponding author: jgpark10@snu.ac.kr

Figure S1. TEM images of Fe_3O_4 NPs with various sizes and SEM image of Fe_3O_4 bulk standard.

Figure S2. Histograms for size distribution of the NPs. For each panel, statistics were calculated with the sample size of 40-60 NPs.

Figure S3. Magnetization curves of 40 nm Fe_3O_4 NPs with different heating/cooling rates. All measurements are done at an external field of 100 Oe and magnetization is normalized.

Figure S4. Magnetization curves of 7 μm and 16 nm samples without oxidation and after oxidation. All measurements are done at an external field of 100 Oe.

Figure S5. Multiple peak fitting results of RIXS spectra shown in Fig. 3c. Symbol mark is experimental data, red line multiple peak fitting result, and blue area extracted low energy excitation peak.

Figure S6. $M(H)$ curves of Fe_3O_4 NPs and bulk standard. Blue and red symbols indicate the data measured at 300 K and 20 K, respectively.

Figure S7. Mössbauer spectra of Fe_3O_4 nanoparticle with the size of 42 nm and bulk sample with the size of 7 μm.

Table S1. Mössbauer data for Fe_3O_4 nanoparticle with the size of 42 nm and bulk sample with the size of 7 μm.

Scheme S1. Schematic illustration of the kinetics of phase transition with increasing particle size.

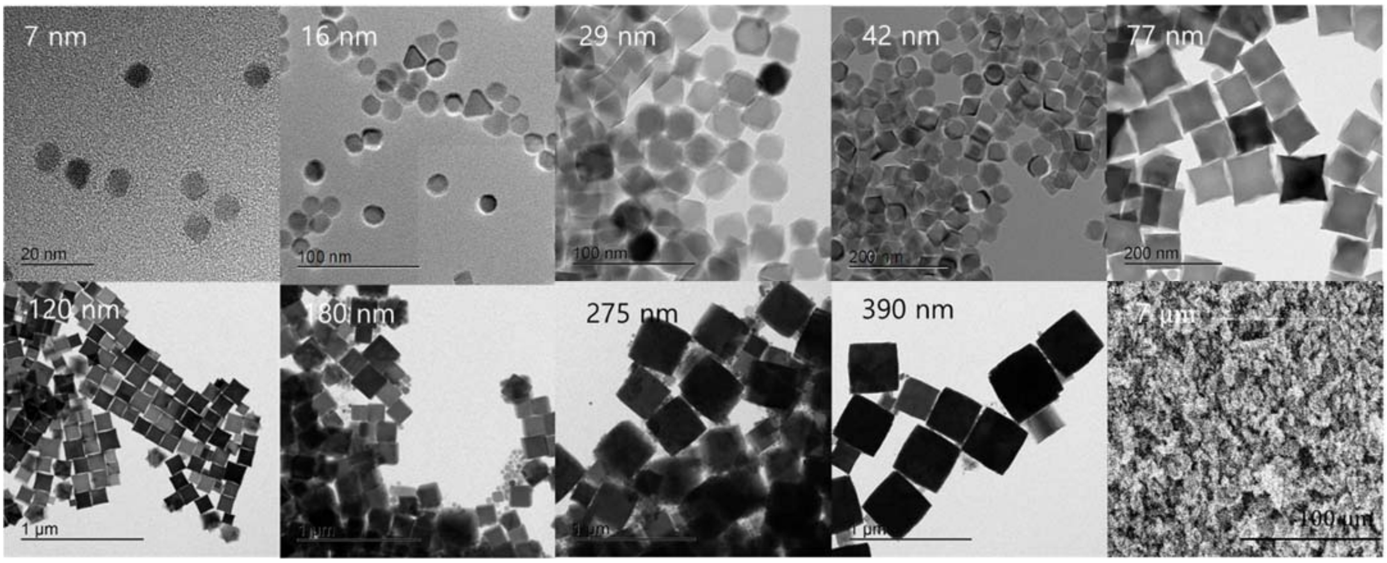

Supplementary Figure S1 **Transmission Electron Microscopy (TEM) images of Fe_3O_4 nanoparticles (NPs) with various sizes and Scanning Electron Microscope (SEM) image of Fe_3O_4 bulk standard.**

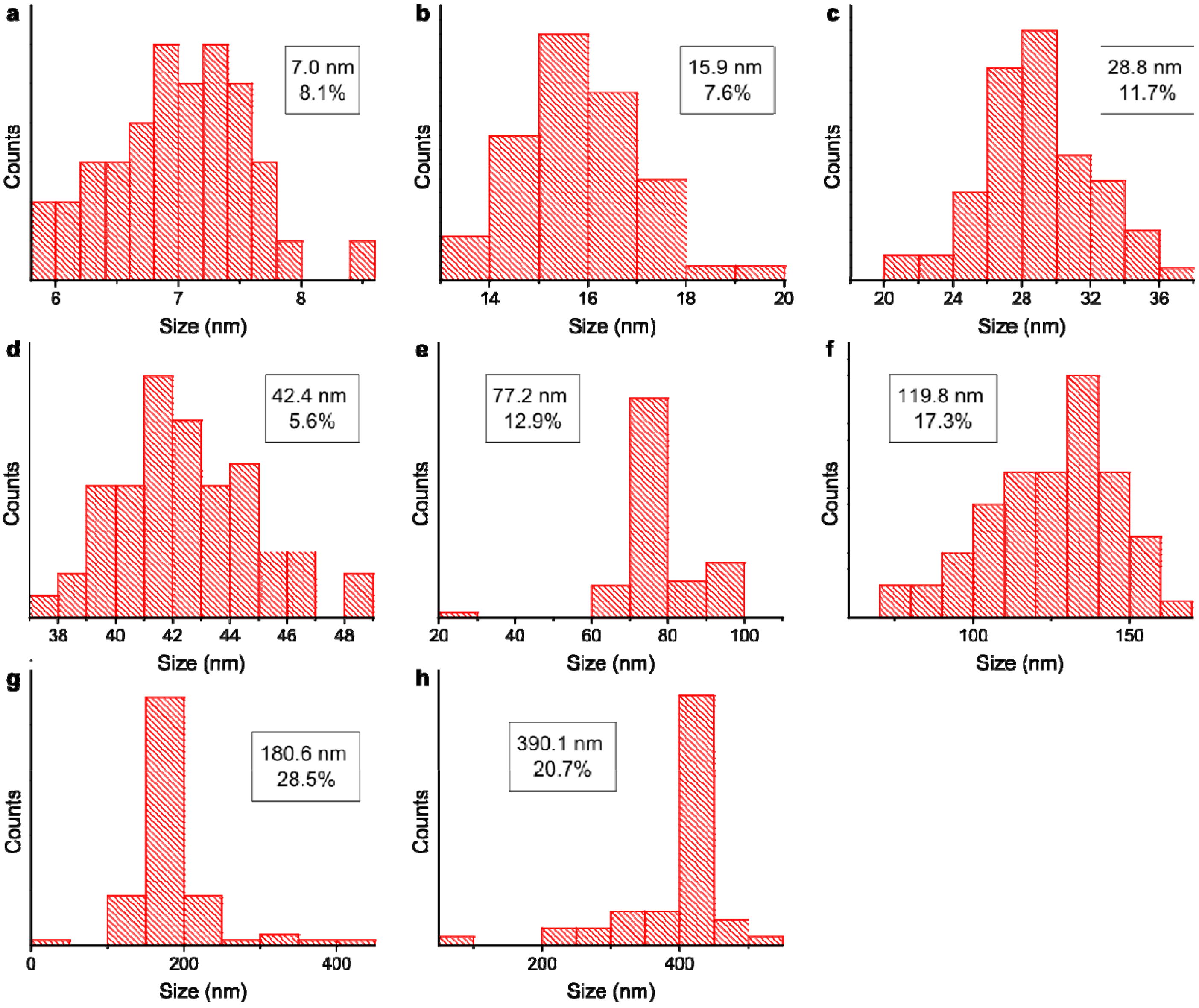

Supplementary Figure S2 **Histograms for size distribution of the NPs.** (a)-(h) For each panel, statistics was calculated with the sample size of 40-60 NPs.

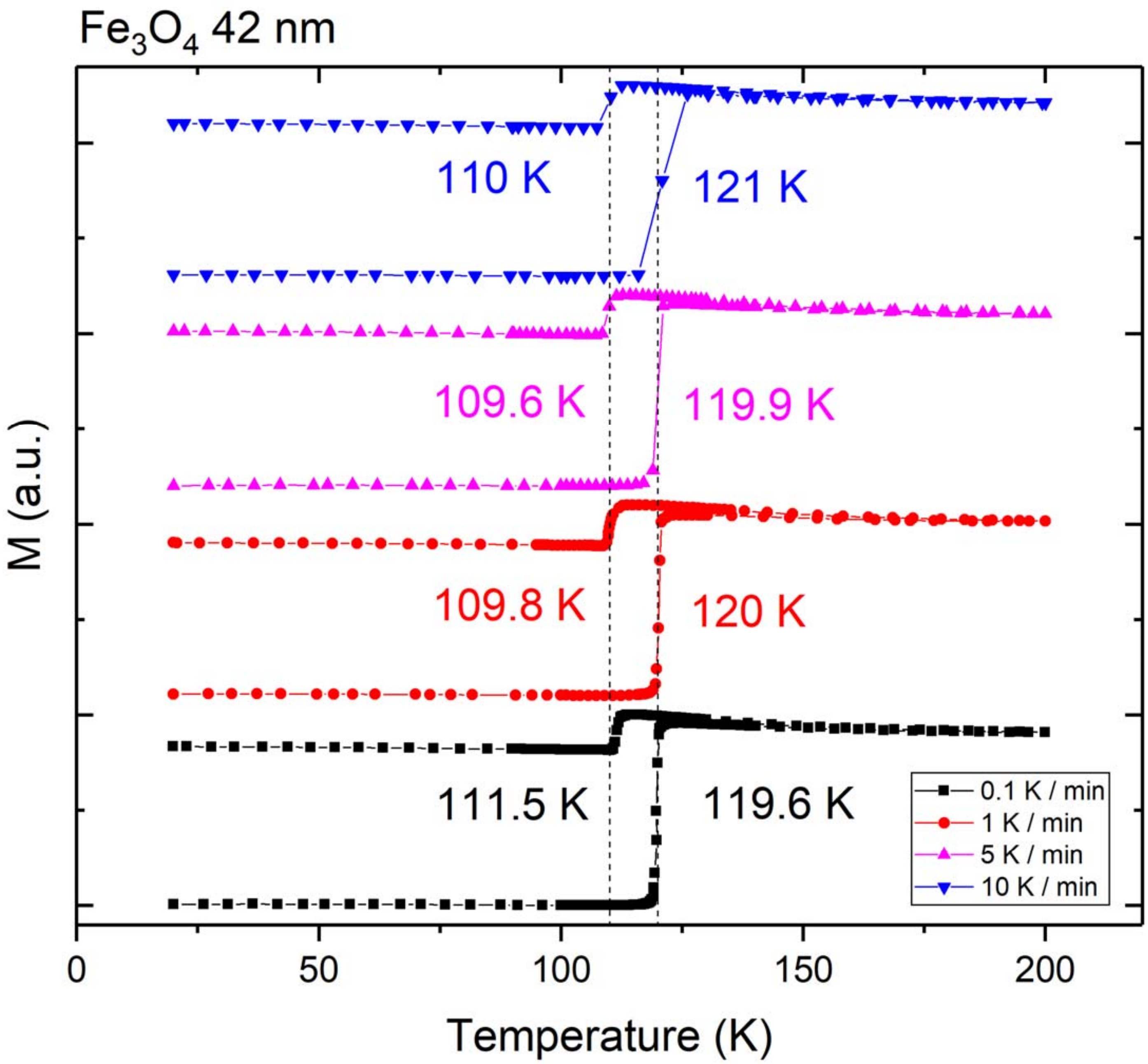

Supplementary Figure S3 **Magnetization curves of 42 nm Fe_3O_4 NPs with different heating/cooling rates.** Four different heating/cooling rates which range from 0.1 to 10 K min^{-1} were selected as shown in the legend of Fig. S3. Both Verwey transition temperature (T_V) and hysteresis width from heating/cooling curves didn't change within error bars. All measurements are done under an external field of 100 Oe.

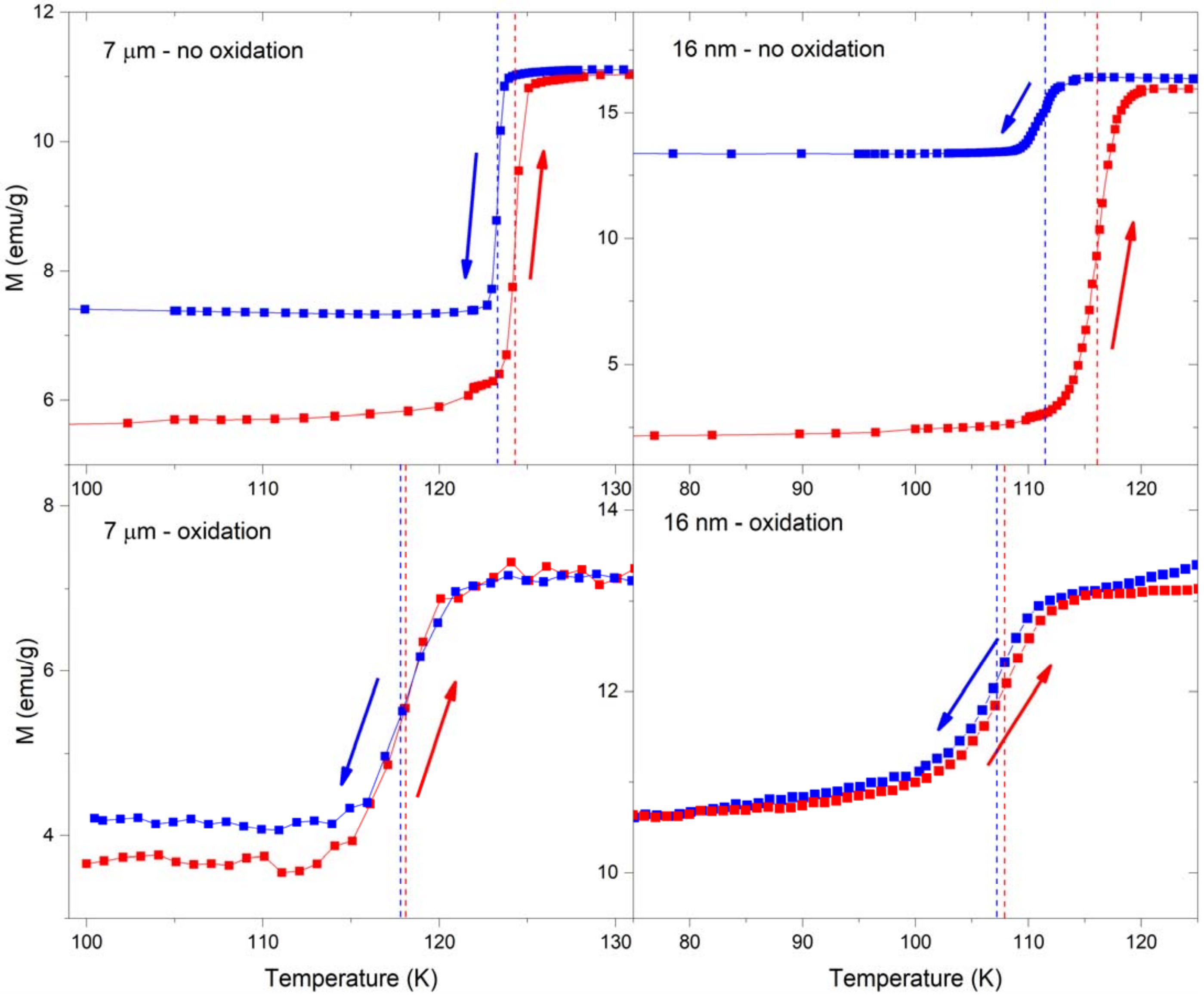

Supplementary Figure S4 **Magnetization curves of 7 μm and 16 nm samples after oxidation.** 7 μm bulk standard sample was oxidized under air at 200 °C for six days and 16 nm samples in ambient air at room temperature for four days. T_v decrease for both samples similar result with the previous study on off-stoichiometry effect[1]. Thermal hysteresis of bulk standard (7 μm) sample change from 1 to 0.3 K and that of 16 nm sample was reduced from 4.6 to 0.7 K.

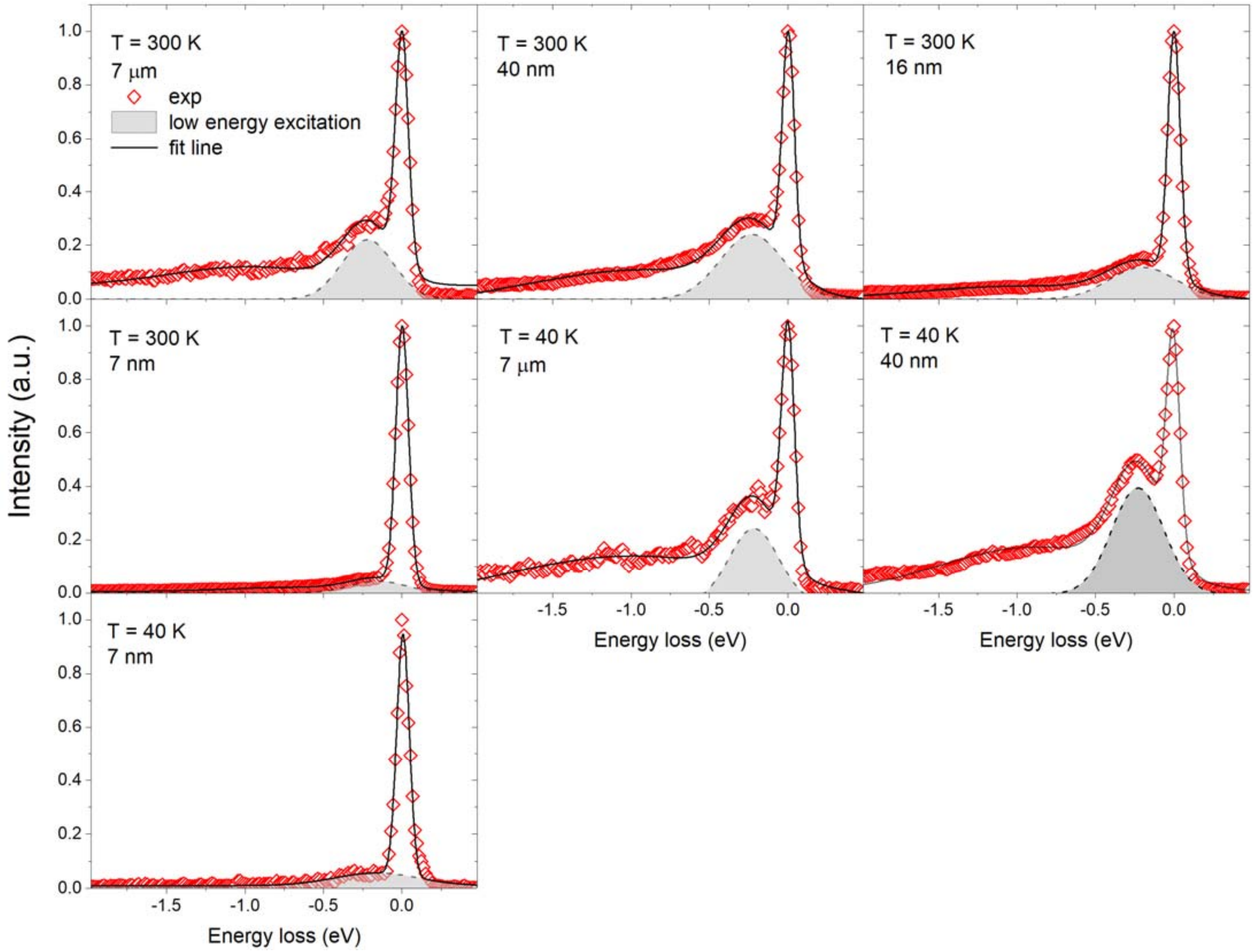

Supplementary Figure S5 **Multiple peak fitting results of Resonant Inelastic X-ray Scattering (RIXS) spectra shown in Fig. 3c.** Symbol mark is an experimental data, red line is a multiple peak fitting result, and blue area is an extracted low energy excitation peak. To fit the spectra, we used three Gaussian functions for elastic peak, low energy excitation at around 0.2 eV and broad hump centered near 1.0 eV.

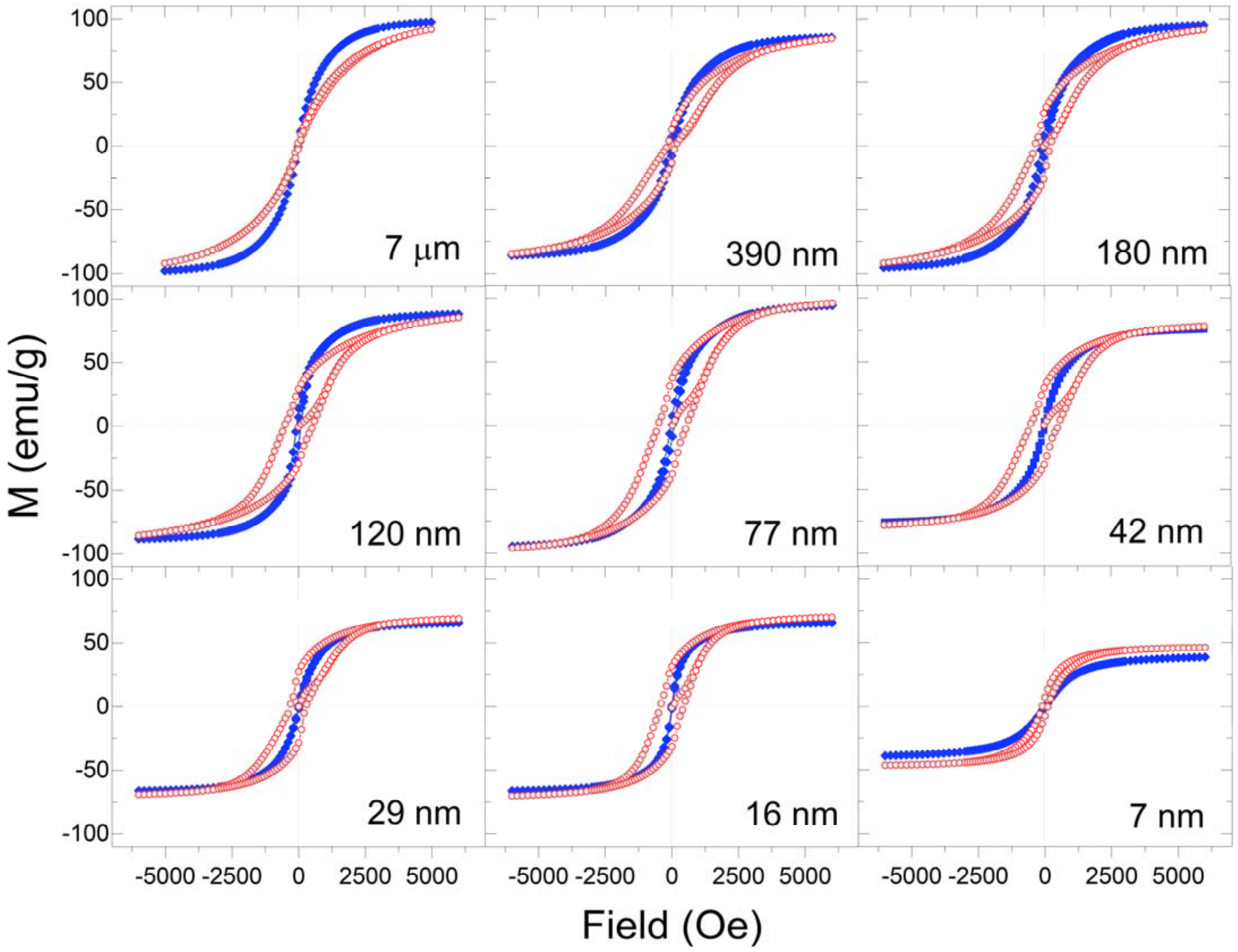

Supplementary Figure S6 ***M(H)* curves of Fe_3O_4 NPs and bulk standard.** Blue and red symbols indicate the data measured at 300 K and 20 K, respectively.

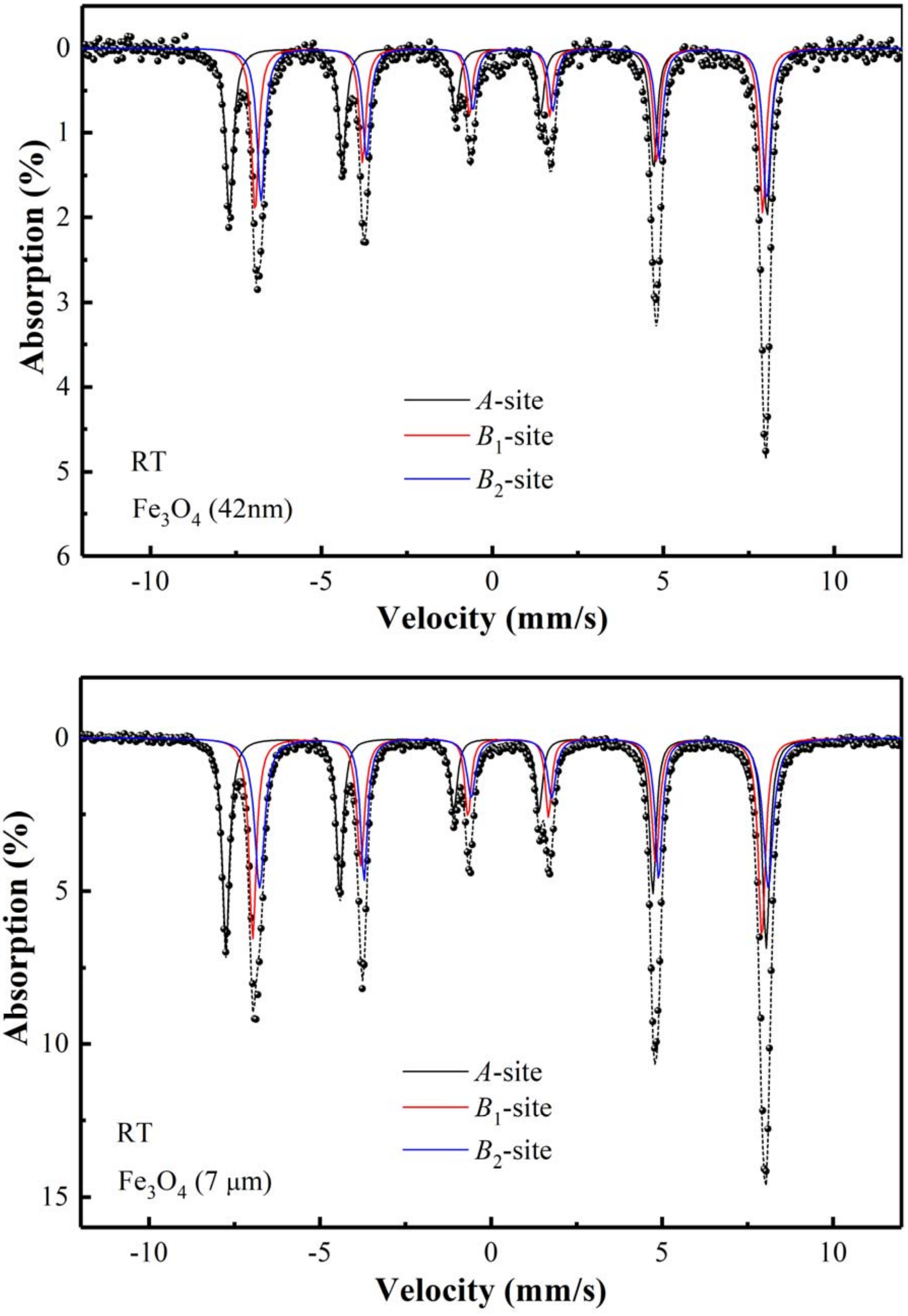

Supplementary Figure S7 ***Mössbauer spectra of Fe_3O_4 nanoparticle with the size of 42 nm and bulk sample with the size of 7 μm.*** The spectra were recorded at room temperature (RT) of 295 K. The black circle is the experimental data and the black dashed line is the total fitting line. The black, red and blue solid lines are the fitting line which represent the tetrahedral A (Fe^{3+}), octahedral B_1 (Fe^{3+}) and B_2 (Fe^{2+}) site of Fe_3O_4 cubic inverse spinel structure, respectively.

Size	Site	H_{hf} (kOe)	E_Q (mm/s)	Δ (mm/s)	*A* (%)
42 nm	A(Fe^{3+})	488.17	0.00	0.16	35.23
	B_1(Fe^{3+})	460.95	-0.01	0.49	31.42
	B_2(Fe^{2+})	459.17	0.01	0.61	33.35
7 μm	A(Fe^{3+})	490.47	0.00	0.15	34.06
	B_1(Fe^{3+})	462.20	-0.01	0.48	32.60
	B_2(Fe^{2+})	461.76	0.04	0.63	33.34

Supplementary Table S1 ***Mössbauer data for Fe_3O_4 nanoparticle with the size of 42 nm and bulk sample with the size of 7 μm.*** H_{hf} = Magnetic hyperfine field, E_Q = Electric quadrupole shift, Δ = Isomer shift and A = Relative absorption area ratio. The errors of each parameter are ±1.00 kOe for H_{hf}, ±0.001 mm/s for E_Q and Δ and ±0.01 % for A.

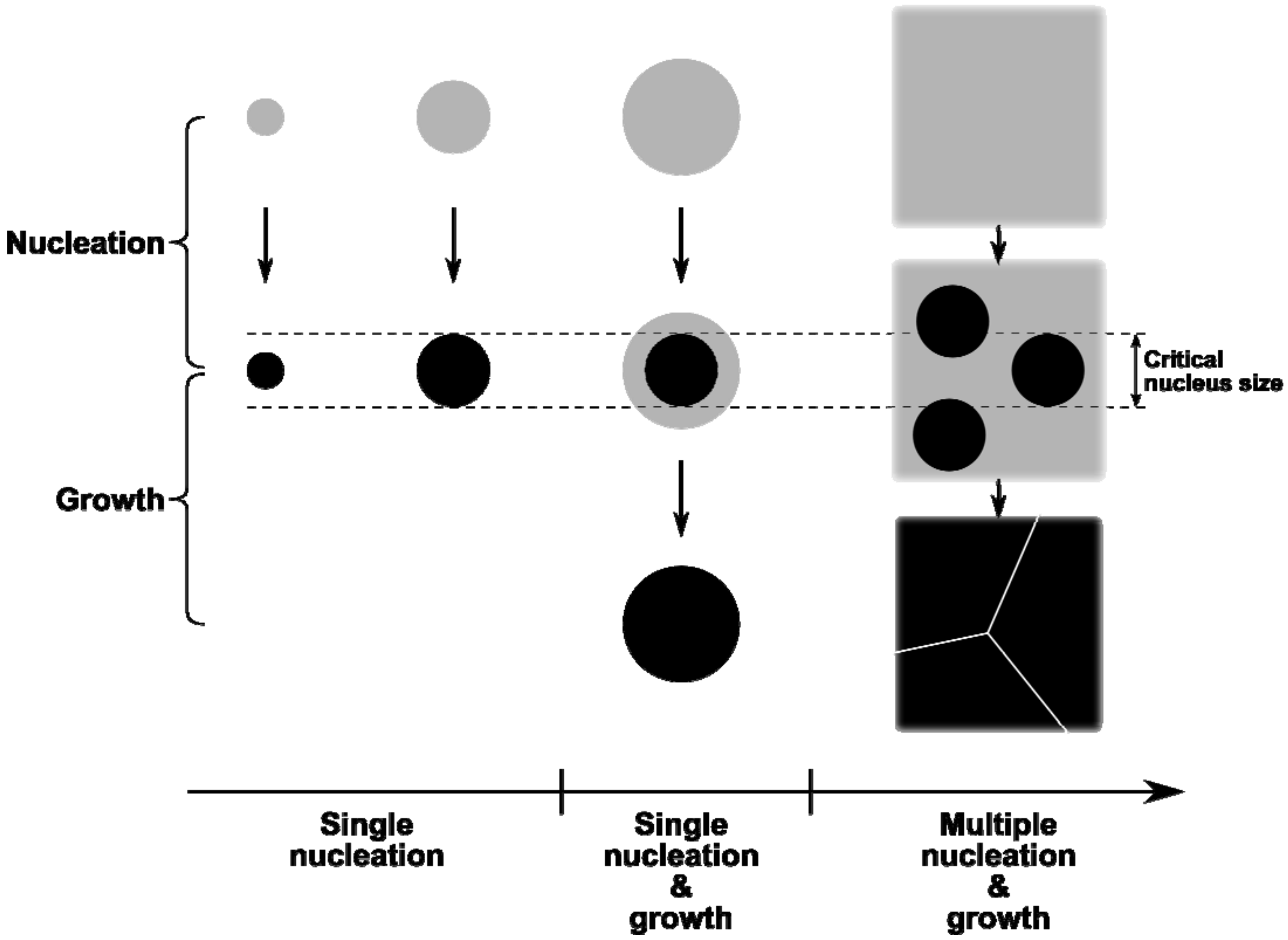

Supplementary Scheme S1 **Schematic illustration of the kinetics of phase transition with increasing particle size[2].**

References

1. Shepherd, J. P., Koenitzer, J. W., Aragón, R., Spałek, J. & Honig, J. M. Heat capacity and entropy of nonstoichiometric magnetite $Fe_{3(1-\delta)}O_4$: The thermodynamic nature of the Verwey transition. *Phys. Rev. B* **43**, 8461-8471 (1991).

2. Chen, C.-C., Herhold, A. B., Johnson, C. S. & Alivisatos, A. P. Size dependence of structural metastability in semiconductor nanocrystals. *Science* **276**, 398-401 (1997).
